# Aggressive mammary carcinoma progression in Nrf2 knockout mice treated with 7,12-dimethylbenz[*a*]anthracene

**DOI:** 10.1186/1471-2407-10-540

**Published:** 2010-10-08

**Authors:** Lisa Becks, Misty Prince, Hannah Burson, Christopher Christophe, Mason Broadway, Ken Itoh, Masayuki Yamamoto, Michael Mathis, Elysse Orchard, Runhua Shi, Jerry McLarty, Kevin Pruitt, Songlin Zhang, Heather E Kleiner-Hancock

**Affiliations:** 1 Department of Pharmacology, Toxicology and Neuroscience, LSUHSC-S, Shreveport, Louisiana, USA; 2 Breast Cancer Focus Group, Feist-Weiller Cancer Center, Shreveport, Louisiana, USA; 3 Center for Advanced Medical Research, Hirosaki University School of Medicine, Hirosaki, Japan; 4 Department of Medical Biochemistry, Tohoku University Graduate School of Medicine, Sendai, Japan; 5 Department of Anatomy & Cellular Biology, LSUHSC-S, Shreveport, Louisiana, USA; 6 Department of Veterinary Medicine, LSUHSC-S, Shreveport, Louisiana, USA; 7 Department of Medicine, LSUHSC-S, Shreveport, Louisiana, USA; 8 Department of Molecular & Cellular Physiology, LSUHSC-S, Shreveport, Louisiana, USA; 9 Department of Pathology, LSUHSC-S, Shreveport, Louisiana, USA

## Abstract

**Background:**

Activation of nuclear factor erythroid 2-related factor (Nrf2), which belongs to the basic leucine zipper transcription factor family, is a strategy for cancer chemopreventive phytochemicals. It is an important regulator of genes induced by oxidative stress, such as glutathione S-transferases, heme oxygenase-1 and peroxiredoxin 1, by activating the antioxidant response element (ARE). We *hypothesized *that (1) the citrus coumarin auraptene may suppress premalignant mammary lesions via activation of Nrf2/ARE, and (2) that Nrf2 knockout (KO) mice would be more susceptible to mammary carcinogenesis.

**Methods:**

Premalignant lesions and mammary carcinomas were induced by medroxyprogesterone acetate and 7,12-dimethylbenz[a]anthracene treatment. The 10-week pre-malignant study was performed in which 8 groups of 10 each female wild-type (WT) and KO mice were fed either control diet or diets containing auraptene (500 ppm). A carcinogenesis study was also conducted in KO vs. WT mice (n = 30-34). Comparisons between groups were evaluated using ANOVA and Kaplan-Meier Survival statistics, and the Mann-Whitney U-test.

**Results:**

All mice treated with carcinogen exhibited premalignant lesions but there were no differences by genotype or diet. In the KO mice, there was a dramatic increase in mammary carcinoma growth rate, size, and weight. Although there was no difference in overall survival, the KO mice had significantly lower mammary tumor-free survival. Also, in the KO mammary carcinomas, the active forms of NF-κB and β-catenin were increased ~2-fold whereas no differences in oxidized proteins were observed. Many other tumors were observed, including lymphomas. Interestingly, the incidences of lung adenomas in the KO mice were significantly higher than in the WT mice.

**Conclusions:**

We report, for the first time, that there was no apparent difference in the formation of premalignant lesions, but rather, the KO mice exhibited rapid, aggressive mammary carcinoma progression.

## Background

Breast cancer is the most common cancer among American women, except for skin cancers. The chance of developing invasive breast cancer at some time in a woman's life is about 1 in 8 (12%). In 2009, an estimated 192,370 new cases of invasive breast cancer will be diagnosed among women in the United States [1]. Many risk factors have been attributed to breast cancer occurrence. Genetic susceptibility accounts for only ~10% of human breast cancer [1,2]. Known environmental risk factors include radiation, obesity, and alcohol use [1,2].

Nuclear factor erythroid 2-related factor (Nrf2) is an important regulator of genes induced by oxidative stress, such as glutathione S-transferases (GSTs), heme oxygenase-1 (HO-1) and peroxiredoxin 1, by activating the antioxidant response element (ARE) [3]. Reactive oxygen species (ROS) are known to activate oncogenic transcription factors such as AP-1 and NF-κB that have been shown in mouse models to be required for carcinogenesis [3]. It has also been noted that Nrf2 deficiency in mice shows an increased risk of chemical carcinogenesis and Nrf2 loss may contribute to tumorigenesis [3]. However, the effects of Nrf2 deficiency in breast cancer have not yet been explored.

Many cancer chemopreventive agents, in particular those that are naturally occurring, boost cellular antioxidant defenses [4]. Evidence is mounting that many of these phytochemicals activate the ARE through Nrf-2. Orally administered AUR induces GST activities via activation of Nrf2, since these effects were significantly attenuated in Nrf2(-/-) knockout (KO) mice [5]. Based on these studies, we hypothesized that AUR could suppress mammary carcinogenesis via activation of Nrf2/ARE. In summary although many chemopreventive phytochemicals are known to activate cellular antioxidant defenses through the ARE, direct evidence that their chemopreventive effects against mammary carcinogenesis are due to this effect is limited.

Oxidative stress is a condition of increased oxidant production in animal cells characterized by the release of free radicals, resulting in cellular degradation. Oxidative stress resulting from excess ROS and/or deficiencies in antioxidant capabilities may play a role in breast cancer etiology [6]. For example, a growing body of evidence suggests that natural and synthetic estrogens, which are oxidized to form quinones, are involved in breast cancer [7]. In a population-based, case-control study (654 cases, 605 controls), African American women harboring the mitochondrial DNA G10398 polymorphism exhibited an increased risk of invasive breast cancer (OR 1.60; 95% CI, 1.10-2.31, P = 0.013) [8]. MtDNA G10398A may be involved in altered structure of Complex I, which could lead to increased ROS [8]. Recently, a nested case-control study of postmenopausal women reported that women with genetic polymorphisms of Nrf2, NAD(P)H quinone oxidoreductase (NQO1), and HO-1 that favored iron-generated oxidative stress were at higher risk of breast cancer [6] An epidemiological study by Santella and colleagues found that women with higher plasma levels of oxidative protein damage (i.e. protein carbonyls) were at higher risk for breast cancer [9]. These studies suggest a role of oxidative stress in breast cancer that may be attenuated by chemopreventive agents.

In order determine whether phytochemicals can prevent mammary carcinogenesis via activation of Nrf2/ARE, first it must be demonstrated that Nrf2/ARE is involved in mammary carcinogenesis. To our knowledge, no mammary carcinogenesis studies have ever been done in Nrf2 knockout mice. Thus, to address this critical gap in knowledge, we conducted a mammary carcinogenesis study in Nrf2 knockout mice. **We report, for the first time, that there was no apparent difference in the formation of premalignant lesions, but rather, the KO mice exhibited rapid, aggressive mammary carcinoma progression. Many questions remain as to the mechanism, which we will explore in future studies**.

## Methods

### Auraptene

Auraptene (AUR), a natural product isolated from citrus, was initially purchased from LKT laboratories, Inc. (West St. Paul, MN) for the previous studies and for the comparison study (see below). In this regard, due to the large quantity of AUR needed to mix in the diet for the premalignant study, it became necessary to synthesize AUR.

### Synthesis of Auraptene

The synthesis of AUR was based on a literature procedure [10], modified as follows. Geranyl chloride (42 g, 0.24 mol), synthesized as reported elsewhere [11], was added to a 2-L round bottom flask along with acetone (800 mL), potassium carbonate (45 g, 0.32 mol), and 7-hydroxycoumarin (35.8 g, 0.22 mol), obtained from Sigma-Aldrich. The reaction mixture was allowed to stir and heated at a gentle reflux under an argon atmosphere for about 3 days, when thin layer chromatography (TLC) showed that no more 7-hydroxycoumarin was present. The reaction mixture was then cooled, filtered, and evaporated to dryness. The residue was dissolved in ethyl acetate and filtered. Removal of the solvent gave crude product (65 g), which was heated in hexanes along with 2 g of activated charcoal. After the product had dissolved, the mixture was filtered hot. The crystals that formed as the mixture cooled were filtered, dissolved in a toluene, hexanes solvent mixture and subjected to flash chromatography (7:1 toluene, hexanes). After collecting and combining the appropriate fractions, the solvent was removed, and the residue was crystallized from hexanes to give ~30 g of product. The ^1^H and ^13^C NMR spectra matched those of the authentic sample. The compound also co-eluted on TLC with the authentic sample. The synthesis of auraptene was carried out by Dr. William H. Johnson, Jr. in the laboratory of Dr. Christian P. Whitman (The University of Texas at Austin). Hence we named the synthetic AUR as "UT-AUR".

### Comparison study: LKT-AUR vs. UT-AUR

We conducted a comparison study to determine if the synthetic AUR (UT-AUR) possessed the same activities as the AUR purchased from LKT (LKT-AUR). This study was conducted exactly the same way as the original study in which we showed that AUR could significantly increase liver cytosolic GST activities in Nrf2 WT but not KO mice [5]. The results of the study were consistent with our previous study, and the synthetic AUR possessed the same activity as that from LKT (Additional File 1: Figure S1). UT-AUR was then shipped to Dyets, Inc. (Bethlehem, PA) where it was mixed with AIN76A and formed into pellets (formulation sheet available upon request). These pellets were used for the AUR arms of the premalignant study.

### Animals

Wild-type (WT) and Nrf2(-/-) knockout (KO) mice on an ICR background were bred and genotyped in our facility for the project. They were housed in a temperature and humidity controlled AAALAC accredited facility under a normal 12-hour light/dark cycle. LSUHSC Institutional Animal Care and Use Committee approved the procedures in accordance with NIH guidelines. Mice were allowed access to food (AIN-76A) and water *ad libitum*. Female mice at 5-6 weeks of age were used for the studies below. The carcinogenesis models described below were adapted according to Aldaz & co-workers [12]. Body weights for the premalignant study and the tumor study are shown in Additional File 1: Figures S3, S4, and S5. Overall, there were no dramatic effects on body weights by any of the treatments.

### Premalignant Study

A 10-week pre-malignant breast cancer study was performed in which 8 groups of 10 each female WT and Nrf2 KO mice were fed either control diet (AIN76A, Dyets Inc. Bethlehem, PA) or diets containing AUR (500 ppm). Mice were shaved on the dorsal flank with clippers and those mice that were to be dosed with carcinogen were first injected with medroxyprogesterone acetate (15 mg, *sc*, purchased from Pfizer Inc., NY, NY). This was considered day 1 of the study. One week later, mice were then administered vehicle (0.1 mL/25 g bw corn oil, negative controls) or dosed with and 7,12-dimethylbenz[a]anthracene (DMBA, 40 mg/kg bw, oral gavage, once/wk for 4 consecutive weeks, purchased from Sigma-Aldrich) (groups labeled as "DMBA") to induce premalignant lesions. All treatments with DMBA were conducted in a light-protected biosafety cabinet, since DMBA is hazardous and light-sensitive. At the end of the 10 weeks, mice were euthanized by CO_2 _asphyxiation in a pre-charged chamber, and mammary glands were collected. Mammary fat pads were also fixed in 10% neutral buffered formalin overnight, then treated with graded alcohols followed by paraffin-embedding, sectioning at 4 μm, and H&E staining. Livers were collected for GST activity assays. Additional mammary fat pads were snap-frozen in liquid nitrogen and stored at -80°C for later analysis. Snap-frozen mammary epithelial tissue was isolated from mammary glands using hyaluronidase and collagenase as previously described [13].

#### Tumor Study

Groups of 10 mice each (WT & KO) were administered vehicle for the negative controls. Groups of 34 mice (WT) and 30 mice (KO) were dosed with medroxyprogesterone acetate and DMBA exactly as was done in the premalignant study. Mice were palpated twice a week for tumors, and body weights were recorded weekly. Tumors were measured using digital calipers and tumor volume (mm^3^) was calculated using the following formula, based on the assumption of a near-spherical tumor shape: V = ((*l *+ *w*)/4)^3 ^* 1.33 * PI, where *l *= length, *w *= width. Necropsies were planned as soon as a mouse appeared moribund or the tumor volume was ≥ 1800 mm^3^. Mice were euthanized via CO_2 _asphyxiation in a pre-charged chamber, and necropsied immediately. Criteria for euthanasia were based, not only on body weight loss or coat condition, but on overall sickness behavior and body condition, described recently by Paster and colleagues [14]. Individual mice were meticulously tracked throughout the entire study by use of ear tags. Data for every individual mouse was recorded on a chart, including the location of the tumor and any other observations, such that the histopathology analyses could be matched to the individual tumor history (growth rate over time, etc.). Necropsies were performed on all mice. Anything that looked like a tumor anywhere in the body was taken for histology, as well as spleens, livers, mammary fat pads, lungs, and occasionally bone and brain to check for metastases. Tumors were verified by two independent pathologists as mammary carcinomas.

#### Western blot analysis

Mammary carcinomas previously snap-frozen in liquid nitrogen and stored at -80°C were pulverized in a mortar and pestle in liquid nitrogen over dry ice. Tissue was never allowed to thaw. Approx. 100 mg of pulverized tissues were lysed in RIPA buffer, further processed, and normalized by protein concentration. Protein concentration was estimated using the bicinchoninic acid method comparing to a BSA standard curve (Thermo Scientific, Rockford, IL). Oxyblot: samples were derivatized using 2,4-dinitrophenylhydrazine as described by Levine & colleagues [15], resolved on a polyacrylamide gel then electroblotted onto a membrane filter (PVDF), and probed for DNP-ylated proteins (indicating protein carbonyls). This procedure was conducted with the aid of the OxyBlot™ Protein Oxidation detection kit from Millipore (Temecula, CA) following manufacturer's instructions. NF-κB: Samples were probed with anti-phospho-Ser^529^-p65 NF-κB antibody (SA Biosciences, Inc.), and detected with enhanced chemiluminescence. Proteins were also detected by a reversible total protein stain as a loading control (G Biosciences, St. Louis, MO). β-catenin: Protein extracts were subjected to polyacrylamide gel electrophoresis using the 4-12% NuPAGE gel system (Invitrogen), transferred to PVDF (Millipore) membranes and immunoblotted. Antibodies included active β-catenin (05-665) & total β-catenin (06-734) from Upstate and β-actin (sc-47778) from Santa Cruz Biotech. Membranes were probed with HRP-conjugated secondary antibodies for one hour. Membranes were washed with TBST and deioninzed water prior to visualization by enhanced chemiluminescence (Pierce). Films for each respective blot were scanned and band intensity was evaluated using Image-J software.

#### Histopathology

Mice were injected with 2-Bromodeoxyuridine (BrdU, 50 mg/kg ip) 2 h prior to sacrifice for the premalignant study. Whole mounts of mammary fat pads were stained with alum carmine for morphometric analysis as previously described by Russo & colleagues [16]. Formalin-fixed, paraffin embedded sections were also cut to 5 μ and stained with H&E for histopathological analysis as described [16]. Cell proliferation was detected by positive nuclear immunohistochemical staining for BrdU in the premalignant study.

### Liver cytosolic GST assays

All samples were homogenized in 0.05 M Tris, pH 7.4 buffer containing 0.25 M sucrose, spun at 10,000 × g, and then the supernatant was transferred into a new tube and centrifuged at 100,000 × g for 60 minutes at 4°C. Bradford Protein Assay performed to estimate protein concentration using BSA as a standard. GST activities were determined using 1-chloro-2,4-dinitrobenzene (CDNB) and 1,2-dichloro-4-nitrobenzene (DCNB) as substrates [5,17]. Both CDNB and DCNB were purchased from Acros (NJ). Briefly, liver cytosolic samples were analyzed using a spectrophotometer reading 340 and 345 nm, respectively, and a cell temp set at 25°C, a lag time of 10 seconds and a rate time of 60 seconds. Caution was given to minimize delay between preparing samples and loading. Activities were calculated using the extinction coefficient of 9.6 mM^-1^cm^-1 ^and 8.5 mM^-1^cm^-1^, respectively.

### Quantitative real-time pcr array

Mammary carcinomas snap-frozen in liquid nitrogen were ground in a mortar and pestle over liquid nitrogen. ~30 mg of powdered tissue was then homogenized and sonicated in lysis buffer containing 2-mercaptoethanol, then subject to RNA isolation and DNase digestion using a mini-spin kit (GE Healthcare). Quantity, purity, and integrity of RNA isolates were verified using Agilent Analysis. Complementary DNA was synthesized from RNA in a thermocycler using qScript™ cDNA SuperMix (Quanta Biosciences, Inc.) according to manufacturer's directions. Finally, cDNA was mixed with the SYBR green master mix (PerfeCTa™ SYBR^R ^Green FastMix™ for iQ™, Quanta Biosciences, Inc.) and loaded onto the Lonza Mouse Stress Response 96 StellARray™ on a BioRad iCycler. Data were analyzed using the Global Pattern Recognition Analysis Tool version 2.0 http://array.lonza.com/apps/gpr/.

### Statistics

Comparisons between WT and KO mice were made using ANOVA followed by Tukey's post-hoc test for incidences of pathologies including tumors, GST activities, tumor weights, and western blots. The Mann-Whitney U-test (non-parametric) was used for tumor multiplicities. Kaplan-Meier survival statistics were employed. Additional tests are described in the figure legends. For body weight data in the tumor study, Area under the curve (AUC) was calculated for each animal by the trapezoid method. Analysis of variance was used to test for differences in AUC between groups.

## Results

### Premalignant Study

**Figure 1:**
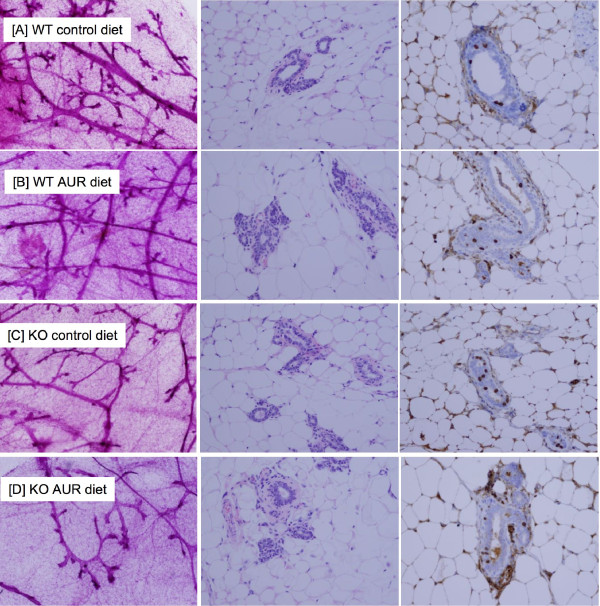
**Representative photomicrographs of the vehicle control groups from the premalignant study**. From L-R: whole mounts (40×, left panels), H&E's (200×, middle panels) and BrdU staining (200×, right panel). Genotype, treatment, and diets are indicated at each row: [A] WT control diet; [B] WT/AUR diet; [C] KO control diet; [D] KO/AUR diet. All photomicrographs were uniformly reduced in size to 70% of the original picture in order to fit into the figure panels. Note, in the BrdU study, some background staining occurred, but this was also detected in the no primary antibody control (data not shown). Nuclear staining of epithelial cells was not observed in the background control.

**Figure 2:**
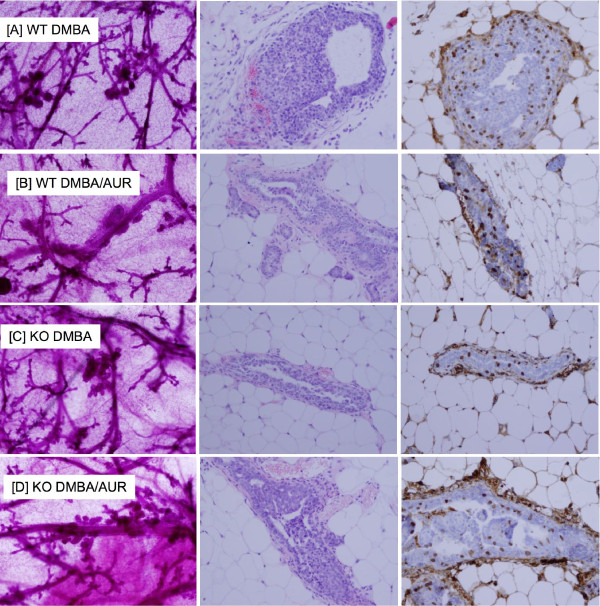
**Representative photomicrographs of the carcinogen-treated groups from the premalignant study**. From L-R: whole mounts (40×, left panels), H&E's (200×, middle panels) and BrdU staining (200×, right panel). Genotype, treatment, and diets are indicated at each row: [A] WT/DMBA control diet; [B] WT/DMBA/AUR diet; [C] KO/DMBA control diet; [D] KO/DMBA/AUR diet. All photomicrographs were uniformly reduced in size to 70% of the original picture in order to fit into the figure panels. Note, in the BrdU study, some background staining occurred, but this was also detected in the no primary antibody control (data not shown). Nuclear staining of epithelial cells was not observed in the background control.

We first conducted a premalignant study, using the protocol described by Aldaz [12]. The duration of the premalignant study was actually determined by the tumor study. In this regard, the tumor study began 3 weeks earlier than the premalignant study. As soon as palpable mammary tumors were observed in the tumor study, the premalignant study was terminated (10 weeks after carcinogen). The results are shown in Figures 1, 2. Analyses of the whole mounts, H&E's, and BrdU incorporation all pointed to the same conclusion. All mice treated with DMBA exhibited a similar degree of premalignant lesions, as characterized by atypical ductal hyperplasia (ADH), and ductal carcinoma in situ (DCIS) (also called early carcinoma) (Figure 2). None of the mice treated with vehicle only developed any of these lesions (Figure 1).

To verify that AUR was inducing GST in WT but not in KO mice as previously observed [5], hepatic GST activities were analyzed. The results are shown in Additional File 1: Figure S2. GST activities using both CDNB and DCNB as substrates were significantly increased in control WT mice fed the AUR diet, but not in control KO mice fed AUR, as expected. In mice treated with DMBA, GST activities were significantly increased in WT mice fed both control and AUR diets using CDNB as a substrate. Thus, it appeared that the DMBA treatment was masking the effect of AUR in the WT mice. In KO mice fed either control or AUR diets, GST activities using DCNB as a substrate were significantly lower than the vehicle control WT mice. Furthermore, the KO mice overall had reduced GST activities compared to WT, regardless of carcinogen treatment or diet. Thus we concluded that the effects of AUR in WT vs. KO mice was consistent with our previous report [5].

The body weight data for the premalignant study is shown in Additional File 1: Figure S3. At day 63 there was no significant difference between the weights of the five groups by ANOVA, p = 0.32. However, on day one there are significant differences by ANOVA, p = 0.002. Two ways of adjusting for this are ANCOVA using day 1 weights as a covariate. In this case the p value for group differences is 0.24, still not significant. The other method is to look at a time-group interaction with repeated measures ANOVA yields p = 0.049, i.e. some groups (the small animals) are gaining faster than the others. However, at day 63 the premalignant weights were similar between groups, p = 0.32. Our overall conclusion is that it was unlikely that any differences in body weight affected our experimental results. It also shows that the AUR diet did not affect body weight, nor did carcinogen exposure.

### Tumor study

**Figure 3:**
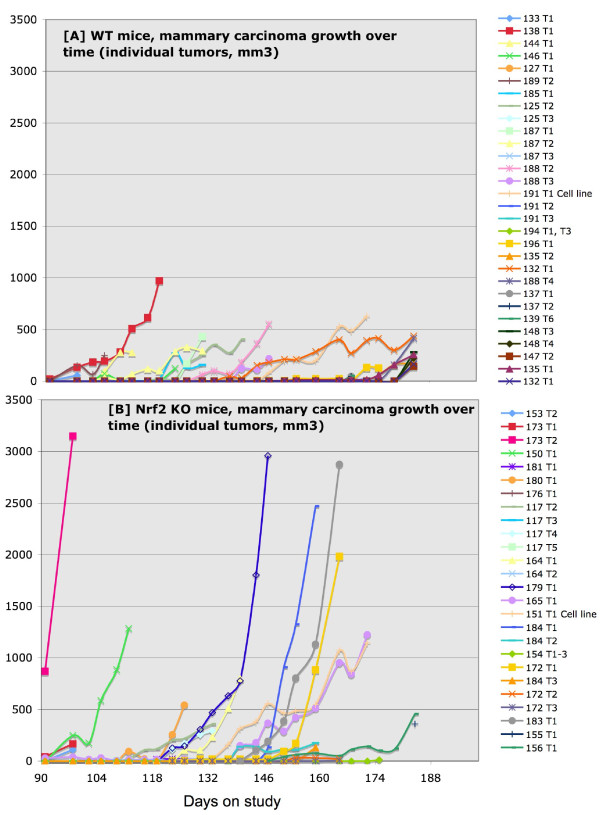
**Mammary Carcinoma Results in Nrf2 WT vs. KO Mice**. [A] Mammary carcinoma growth rate (y-axis denotes tumor volume, mm^3^) in WT mice. [B] Mammary carcinoma growth rate (y-axis denotes tumor volume, mm^3^) in KO mice.

**Figure 4:**
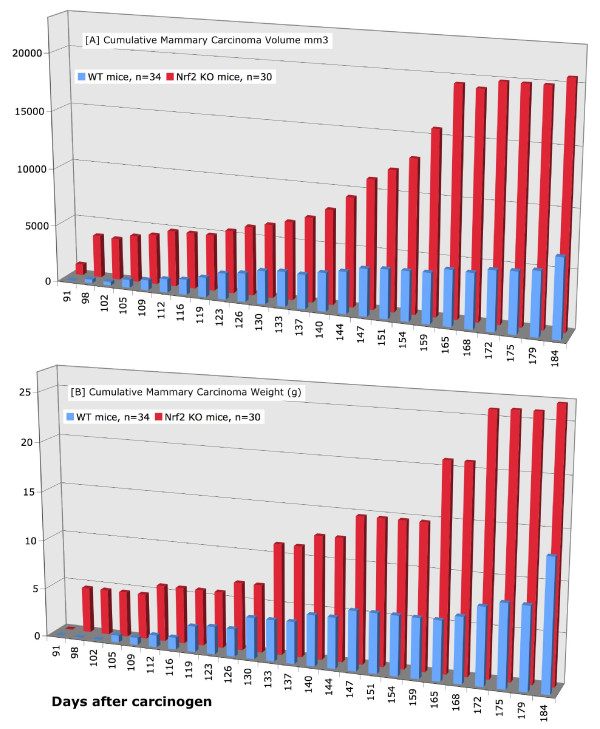
**Mammary Carcinoma Results in Nrf2 WT vs. KO Mice**. [A] Total cumulative volume of palpable mammary carcinomas as measured by calipers (mm^3^). [B] Total cumulative tumor weight of palpable mammary carcinomas as determined at necropsy (g).

**Figure 5:**
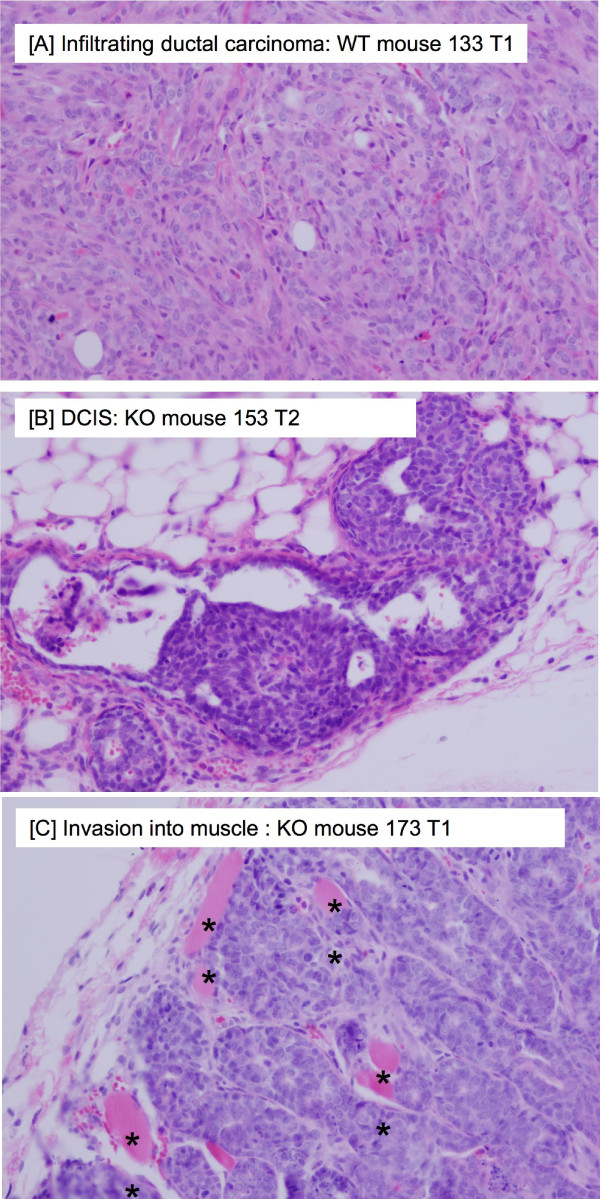
**Histopathology results**. [A] Representative H&E section of WT mammary carcinoma, as reviewed by pathologist. [B] Premalignant lesion (DCIS) in a KO mouse. [C] Photomicrograph of H&E section of KO mammary carcinoma, as reviewed by pathologist. ** denotes invasion into muscle. Magnification × 400.

As mentioned above, the premalignant study began 3 weeks after the tumor study, and we terminated the premalignant study at the first observation of tumors in the tumor study. To our astonishment, there was a dramatic difference in the rate of growth and in the size of the mammary carcinomas. However, there was no difference in mammary carcinoma latency (Table 1). As shown in Figures 3, 4, Table 1, and Additional File 1: Table S6, the Nrf2 KO mammary carcinomas grew at a faster rate (Figure 3), had a significantly larger volume (Figure 4A), and significantly greater tumor weight at necropsy (Figure 4B). In the WT mice, the average rate of mammary carcinoma growth was 28 mm^3^/day (range 8 - 69 mm^3^/day), not including tumors ≤ 0.05 g (Figure 3A, Additional File 1: Table S6). A stable, immortalized cell line has been isolated from WT 191 T1. In the KO mice, the average rate of growth was 96 mm^3^/day (range 13 - 326 mm^3^/day), not including tumors ≤ 0.05 g (Figure 3B and Additional File 1: Table S6). A stable, immortalized cell line has been isolated from KO 151 T1. Tumors were measured by caliper and volume estimated using the formula for a sphere. Only mammary carcinomas as determined by both pathologists are included in these charts. As shown in Figure 4A-B, the caliper data was consistent with tumor weight at necropsy. Aside from mammary carcinoma growth rate and size, there were no other differences in mammary carcinoma latency, incidence, or multiplicity. Photomicrographs of the mammary carcinomas are depicted in Figure 5. In Figure 5B, a premalignant lesion is shown surrounded by adipocytes. As shown in Figure 5C, some of the mammary carcinomas invaded into muscle. However, it was not clear whether there were differences in invasion between WT and KO mice. Thus the main conclusion from the tumor study is that, despite no apparent differences in between WT and KO mice in the premalignant study, the KO mammary carcinomas exhibited rapid, malignant progression.

**Table 1:** Mammary carcinoma summary

Pathology analysis	WT	KO
No. of mice in study	34	30
No. of mice with MC	20	18
Tot. no. of MC's	33	29
Avg no. of MC's/mouse	1.64	1.61
Cumulative total MC wt (g)	13.7	30.4
Average MC wt (g)/mouse	0.68 ± 0.15	1.69 ± 0.35**
MC latency	125 days	121 days

Summary of mammary carcinoma incidence, multiplicity, total cumulative weight, latency, and average tumor weight per mouse. The weights of the mammary carcinomas were significantly higher in KO than WT mice. MC, mammary carcinoma. **p < 0.02.

**Figure 6:**
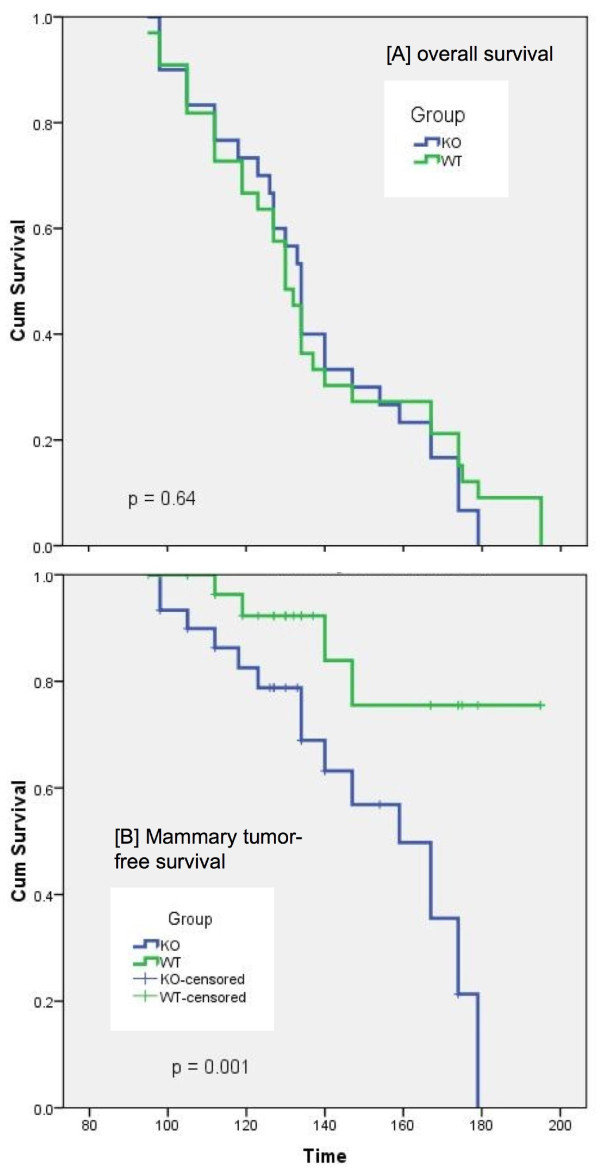
**Kaplan-Meier Survival analysis of [A] overall survival, [B] mammary tumor-free survival**.

Another major difference between the WT and KO mice in the study was the incidence of high-grade lymphomas and cause of death. Although there was no significant difference in the % of mice with any lymphoma, there was a significantly higher incidence of high-grade lymphoma (20% vs. 3% in WT vs. KO mice, respectively, Table 2). When the any lymphoma data was stratified by location, a significantly higher % incidence of spleen with lymphoma involvement was observed (32% vs. 10% in WT vs. KO, respectively). In fact, although there was no difference in overall survival between WT and KO mice (Figure 6A), many of the WT mice had to be euthanized earlier on in the study due to illness caused by lymphoma. However, the p-value for lymphoma-free survival was not significant (p = 0.14). In contrast, more of the KO mice had to be euthanized earlier on in the study due to large palpable mammary tumor volume, most of which were the mammary carcinomas. The mammary tumor free survival was considerably different between the groups (p = 0.001, Figure 6B). Taking into consideration the most likely cause of death, there was a two-fold greater percentage of KO mice that died/were euthanized due to palpable tumor burden (60 ± 9% in KO vs. 29 ± 8% in WT) (Additional File 1: Table S7). We currently cannot explain the striking difference between WT and KO mice in high-grade lymphoma incidence and severity.

**Table 2:** Incidence of lesions in Nrf2 mammary tumor study

Lesion	WT-% inc	KO-% inc	p-value
*Mammary region*			
Hyperplasia	55.9 ± 0.5	46.7 ± 0.5	
Pm (DCIS, early Ca)	67.6 ± 0.5	63.3 ± 0.5	
Mam. carcinoma	58.8 ± 0.5	66.7 ± 0.5	
Sebaceous	2.9 ± 0.2	3.3 ± 0.2	
Adenoacanthoma	5.9 ± 0.2	20.0 ± 0.4	0.091
Lung mets (mc)	5.9 ± 0.2	6.7 ± 0.2	
Sarcoma	8.8 ± 0.3	3.3 ± 0.2	
Other (fibroadenoma, cyst)	8.8 ± 0.3	13.3 ± 0.3	
			
*Lymphomas (by location)*			
Lung	23.5 ± 0.4	16.7 ± 0.4	
Spleen	32.4 ± 0.5**	10.0 ± 0.3	0.031
Liver	29.4 ± 0.5	16.7 ± 0.4	
Mediastinal mass	35.3 ± 0.5	20.0 ± 0.4	
Mammary gland	11.8 ± 0.3	6.7 ± 0.2	
Lymph node	0	6.7 ± 0.2	
Other (internal mass, bone)	0	10.0 ± 0.3	
			
			
Mice with any lymphoma	47.1 ± 0.5	36.7 ± 0.5	
Any high-grade lymphoma	20.6 ± 0.4**	3.3 ± 0.2	0.042
			
*Other lesions*			
Lung adenomas	17.6 ± 0.4**	46.7 ± 0.5	0.012
Skin pap/carc	26.5 ± 0.5	10.0 ± 0.3	0.095
Liver steatosis	23.5 ± 0.4**	3.3 ± 0.2	0.020
Ovarian sex cord stromal	11.8 ± 0.3	6.7 ± 0.2	

Values represent the mean incidence (% Inc.) of lesions ± SD (n = 34 WT, 30 KO). ** Significantly different from KO mice, p ≤ 0.05. Pm, premalignant lesions, mc, mammary carcinoma. P-values are shown for differences that were either significant (p ≤ 0.05) or marginally significant (p ≤ 0.1).

In addition to mammary carcinomas, many other lesions were observed in the study (Table 2). However, we included 10 age-matched control mice per genotype (dosed with vehicle only), and euthanized 1 or 2 at a time with each necropsy. There were no tumors or other abnormalities in any of the control mice. In the mammary region, by thorough evaluation of both whole mounts and H&E's, no difference in the % incidence of hyperplasia or premalignant lesions (DCIS, early carcinoma) was found. Other lesions typically observed in DMBA-treated mice include adenoacanthomas, which often have keratin pearls and other Squamous components [12,18]. These tumors often grew very large as well, and reached up to 20% incidence in the KO mice, but only 6% incidence in the WT mice (p ≤ 0.1). Interestingly, there appeared to be more skin lesions in the WT mice, particularly early on in the study. These included skin papillomas and/or Squamous cell carcinomas. Although the % incidence was 26% in the WT mice and only 10% in the KO mice, these differences were only marginally significant (p ≤ 0.1). Most of the skin lesions occurred on areas of the body in which rubbing occurs, such as the chest, face, forepaws, or next to the ear tag. Again the presence of these lesions was not a surprise because it is known that oral administration of DMBA leads to the formation of epidermal DMBA-DNA adducts [19]. It is also known that wounding of the skin also acts as a tumor promoter [20]. There was a significantly higher incidence of hepatic steatosis in the WT vs KO mice (24% vs 3%, respectively) and the cause of this is unclear. Other lesions included ovarian sex cord stromal tumors, but there was no difference in incidence between WT and KO mice (12% vs. 7%, respectively). Interestingly, the Nrf2 KO mice displayed a significantly higher incidence of lung adenomas (46.7% compared to 17.6%, p = 0.012).

**Figure 7:**
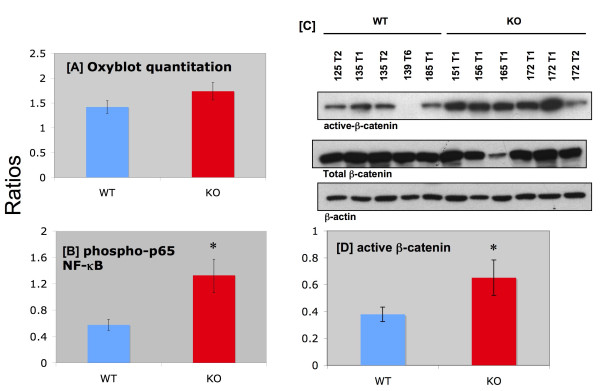
**Western blot analyses of mammary carcinomas**. **Panel A:** Ratio of oxidized proteins: total protein in mammary carcinomas. Values represent means ± SE (n = 7-8). No significant difference was observed between WT and KO. The oxyblot was repeated a second time with additional samples (n = 9-12), and similar results were observed. **Panel B:** Ratio of phospho-p65-NF-κB/total NF-κB (normalized to total protein) in mammary carcinomas. Values represent means ± SE (n = 5-6) of individual tumors. This ratio was significantly higher in the KO mammary carcinomas. * Significant difference (ANOVA p < 0.05). **Panel C:** Representative western blot comparing WT and KO mammary carcinoma expression of β-catenin. Top panel, active β-catenin, middle panel, total β-catenin, lower panel, β-actin (loading control). Tumor lysates from individual mice were loaded on the gel as indicated at top of figure. **Panel D:** Summary of β-catenin results. Figures represent the ratio of active/total β-catenin (means ± SE, n = 9-10). * Significant difference (ANOVA p < 0.05).

Much of the molecular basis for the intriguing difference in WT vs. KO mice in mammary carcinoma progression remains to be determined. However, there was no significant difference in protein carbonyls between WT and KO mammary carcinomas (Figure 7A). Interestingly, there was a ~2-fold increase in the expression of the active form of NF-κB (Figure 7B) in the KO mammary carcinomas. However, a histopathological analysis of the mammary carcinoma H&E's revealed no apparent difference in inflammatory components between WT and KO. The active form of β-catenin was also significantly increased by nearly 2-fold in the KO mammary carcinomas (Figure 7C-D).

**Figure 8:**
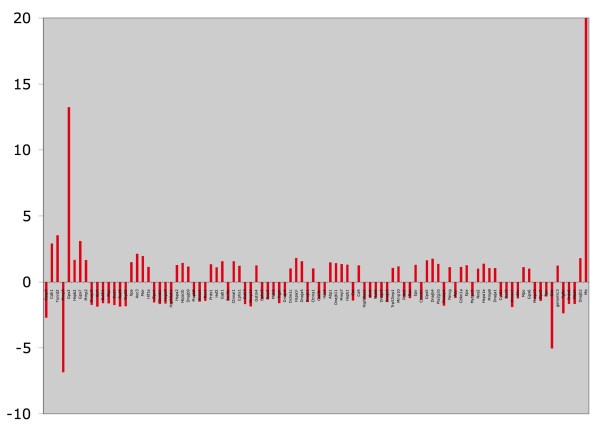
**GPR results for q-rt-pcr Stress Response 96 well array**. KO mammary carcinomas are compared to WT as the control. Figures represent fold-change.

We hypothesized that there could be differences in the stress pathway, so we conducted quantitative real-time pcr on the mammary carcinomas (4 WT and 4 KO, Figure 8 and Table 3). Although there were no significant p-values (likely due to the heterogeneity of these carcinomas), there were several "leads" that are listed in Table 3. These include apparent decreases in caspase-3 and mmp-9, but increases in cadherin 1, thioredoxin reductase 2, and glutathione peroxidase 3 in the KO mammary carcinomas. Although we would expect to see a decrease in caspase-3 (leading to reduced apoptosis in the KO mammary carcinomas), we were surprised to observe the apparent decrease in mmp-9, and higher expressions of antioxidant genes. Without further analysis, we can only speculate that these may be compensatory responses to the tumor microenvironment.

**Table 3:** Comparison of WT vs KO mice Quantitative real-time pcr array: mouse stress response pathway

Rank	Well	Gene Name		Fold Change
1	A10	Casp3	caspase 3, apoptosis related cysteine protease	**-2.69**
2	H02	Cdh1	cadherin 1	**2.91**
3	H12	Txnrd2	thioredoxin reductase 2, mitochondrial	**3.53**
4	A03	Mmp9	matrix metallopeptidase 9	**-6.83**
5	F03	Gpx3	glutathione peroxidase 3	**13.2**
6	C03	Hspb2	heat shock protein 2	**1.66**
7	G03	Gpx7	glutathione peroxidase 7	**3.09**
8	D06	Mmp2	matrix metallopeptidase 2	**1.65**
9	A12	Dnajc5	DnaJ (Hsp40) homolog, subfamily C, member 5	**-1.73**
10	D05	Aprt	adenine phosphoribosyl transferase	**-1.86**
11	B06	Bcl2l1	BCL2-like 1	**-1.59**
12	F10	Myc	myelocytomatosis oncogene	**-1.60**
13	F06	Sod1	superoxide dismutase 1, soluble	**-1.74**
14	E12	Rad9	RAD9 homolog (S. pombe)	**-1.86**
15	C04	Trp53	transformation related protein 53	**-1.82**
16	H08	Xpa	xeroderma pigmentosum, complementation grp A	**1.49**
17	F07	Aoc3	amine oxidase, copper containing 3	**2.13**
18	H10	Pklr	pyruvate kinase liver and red blood cell	**1.96**

Global Pattern Recongition TM (GPR) Results for Mouse Stress Response 96 StellARray™. Four WT mammary carcinomas (125T3, 135T1, 191T3, and 139T6) were compared to four KO mammary carcinomas (183T1, 165T1, 155T1, and 172T1). The selection of mammary carcinomas for the real-time pcr experiment was based on the date of necropsy so that the mice would be fairly well age-matched. The average days on the study for the WT compared to Nrf2 KO mice were 166 vs. 170, respectively. Also, these tumors were strictly mammary adenocarcinomas, not mixed with lymphoma or adenoacanthoma. P-values ranged from 0.11 - 0.24, and so these results are considered "leads" not statistically significant.

The body weights of all mice in the tumor study, including the vehicle control mice, are shown in Additional File 1: Figure S4. There was no overall statistical difference between groups, p = 0.08. The Tukey HSD post-hoc test was used to compare pairs of groups. No significant differences were found, p-values ranged from 0.08 to 0.89 (box plot, Additional File 1: Figure S5). These results suggest that body weight was not a factor in the experimental results, and that carcinogen exposure did not affect body weight. However, mice found to be moribund were euthanized, so it is unlikely that cachexia was involved in the study.

## Discussion

The role of Nrf2 activation in the chemoprevention of many types of cancer has been well-established [21]. However, little is known about the role of Nrf2 in the prevention of mammary carcinogenesis. The current study demonstrated that targeted deletion of Nrf2 in mice treated with DMBA resulted in rapid, aggressive mammary carcinoma growth rate, characterized by significantly larger tumor volume and tumor weight. In contrast, Nrf2 activation (through dietary exposure to AUR) or deletion (KO mice) did not appear to affect the formation of premalignant lesions. Induction of the so-called "phase II detoxifying enzymes" would be expected to block tumor initiation [22], so these results were surprising. On the other hand, we have previously shown that the linear furanocoumarins, such as imperatorin and isopimpinellin, are more effective at suppressing DMBA-DNA adduct formation compared to the simple coumarins, limettin and coumarin [13]. Linear furanocoumarins are much more potent inhibitors of the cytochrome P4501 family of enzymes that bioactivate DMBA [13]. In contrast, AUR and other simple coumarins do not exhibit this effect. Thus, the apparent lack of effect of AUR on premalignant lesions in the current study should not be discouraging. We recently reported that dietary administration of AUR delayed onset of N-methylnitrosourea-induced mammary carcinomas in rats, which corresponded to a decrease in cyclin D1 protein expression [23]. This suggests that AUR affects later stages of mammary carcinogenesis, such as promotion and/or progression.

The model chosen for initiating carcinogenesis was selected based on a report by Aldaz and colleagues that pre-treatment with depot medroxyprogesterone acetate increased DMBA-induced mammary tumor incidence, and significantly shortened the latency [12]. Also there were fewer non-mammary tumor related deaths (8%) in their study compared to the DMBA only groups (51%-81%). In our study, we observed a high level of morbidity and mortality in the WT group (46% due to lymphomas). However, the background strains of the mice were different between our study and theirs (ICR vs. CD2F1, respectively).

Understanding the molecular basis for the drastic differences between mammary carcinogenesis in WT vs. Nrf2 KO mice is critical. The 2-fold increase in NF-κB activation in Nrf2 KO mammary carcinomas was intruiguing and suggests possible cross-talk between Nrf2 and NF-κB. This concept was explored recently by Tusi and colleagues [24], who demonstrated that triazine derivatives, which possess anti-cancer activities, inhibited the activation of NF-κB by H_2_O_2 _in PC12 cells. The triazine derivatives also induced HO-1, induced Nrf2 protein expression, and increased GSH content. The conclusion was that the Nrf2 mediated neuroprotective effects of these triazine derivatives may be due to suppression of NF-κB. Thus, the exact role of NF-κB activation in mammary carcinogenesis in the Nrf2 KO mice remains to be determined.

The increased expression of activated β-catenin in the Nrf2 KO mammary carcinomas was also interesting. It has been previously reported that the heterogeneity of DMBA-induced mouse mammary carcinomas could be due to changes in the Wnt pathway in mammary progenitor cells [18]. Nucleoredoxin is a member of the redox-catalyic family including thioredoxin, which is regulated by Nrf2 activation [25]. Nucleoredoxin acts as a redox-sensor that negatively regulates signaling of Wnt/β-catenin by forming a complex with Dissheveled (DVL) [26]. In the "on state", Wnt binds to its receptor, frizzled (FZD). DVL inhibits the β-catenin destruction complex, thus β-catenin accumulates, translocates to the nucleus, where it activates transcription factors for target genes [26]. Therefore, we hypothesized that the active form of β-catenin would be elevated in the KO mammary carcinomas, which was supported by our data.

In regards to the quantitative real time pcr array, we consider these results to be leads for future studies. We will highlight a few of the top leads herein. The downregulation of Casp3, a pro-apoptotic protein [27], in the KO mammary carcinomas might suggest that the tumors are more resistant to apoptosis, which could explain why the rapid growth and size. However, upregulation of Cdh1 and downregulation of Mmp9 are counter-intuitive to the observed results. Cdh1 regulates cellular adhesion, and loss of Cdh1 is associated with invasion and metastasis [28]. Mmp9 is a gelatinase also involved in tumor invasion [29]. Also, the striking increase in thioredoxin reductase 2 (Txnrd2) and the selenoproteins glutathione peroxidase 3 and 7 (Gpx3, GPX7) may explain why there were no differences in oxidized proteins in WT vs. KO mammary carcinomas. This would then suggest that these genes are regulated by factors other than or in addition to Nrf2. Txnrd2 is a mitochondrial defense against oxidative stress [30]. Gpx3 is expressed in plasma and normal kidney where it counteracts oxidative stress [31]. It can be increased at various stages of cancer, but also possesses protective effects. Gpx are regulated by Nrf2 and p63, which is a member of the p53 family, but not p53 itself [31]. Overexpression of Gpx inhibited p53-dependent H_2_O_2 _induced apoptosis in human MCF-7 breast carcinoma cells. Gpx7 is silenced in Barrett's oesophagus and adenocarcinoma via epigenetic mechanisms [32]. Again at this point we can only speculate that these are compensatory mechanisms as the KO carcinomas adapt to their environment.

In our study, the Nrf2 KO mice also displayed significantly higher incidence of lung adenomas. It is clear from the literature that under certain experimental conditions, phytochemicals and other molecules that activate Nrf2 confer protection from the early stages of carcinogenesis (reviewed in [4,33]). Enzymes that can protect against oxidative stress and detoxify carcinogens are induced, thus protecting the body from harmful chemicals such aflatoxin B1 and benzo[a]pyrene. For example, daily oral dosing of 125 mg oltipraz for a month in humans increased the excretion of aflatoxin-mercapturic acid [34]. In rodents treated with aflatoxin B1, dietary exposure to oltipraz induced phase II carcinogen detoxifying enzymes including GST, and conferred protection against DNA adduct formation and tumorigenicity [35,36]. In another example, dietary exposure to dibenzoylmethane activated Nrf2/ARE and decreased the formation of benzo[a]pyrene DNA adducts in A/J mouse lungs [37]. With these and other examples in the literature, the chemopreventive agents were administered prior to disease progression. Thus it may not be surprising that there were increased lung adenomas in the Nrf2 KO mice treated with DMBA.

On the other hand, epidemiological evidence and xenograft models with lung carcinoma cells have suggested that Nrf2 is upregulated in cancer and that suppression of Nrf2 may be a therapeutic target [38,39]. In consideration of our findings with the published literature, the sequence of Nrf2 activation over time during carcinogenesis may be the key factor in determining whether it is protective or stimulatory in cancer. Whereas antioxidant enzymes may be preventive against cancer development, in already transformed cells, these enzymes may confer protective advantage of cancer cells in the hostile tumor microenvironment [40,41]. In this regard, Nrf2 upregulation of cellular antioxidant defense may protect cancer cells from oxidative stress, and make them more resistant to chemotherapy and radiation. Furthermore, an N-terminal domain mutation of Keap-1 (C23Y) was identified in human breast cancer [42]. Nioi and Nguyen demonstrated that this mutation impairs the ability of Keap-1 to repress Nrf2 activity [43]. However, what is not known is when in the carcinogenic process is Keap-1 is mutated. Carcinogenesis results in compounding mutations, and by end-stage disease, causative factors can be difficult to discern. The potential effects of chemopreventive phytochemicals in cancer patients may not be clear. Recently, a comparison of genetic vs. pharmacologic activation of Nrf2 using Keap-1 knockout mice and a synthetic oleanane triterpenoid, respectively [44]. Distinct but overlapping genetic changes exist, suggests that dietary intervention with phytochemicals may not have the same effect as genetic manipulation. Up to now, most of the Nrf2 manipulations in cancer cells have been done using sh-RNA against already transformed cancer cells. We hope that our future characterization of the stable, immortalized WT and Nrf2 KO mammary carcinoma cell lines we developed will help address the conflicting role of Nrf2, because Nrf2 was either present or knocked-out completely *prior *to cancer development.

In a broader sense, the current results may offer clues into factors that drive rapid malignant progression. These results are consistent with the idea that ROS can act as signaling molecules to redox sensitive pathways [25]. However, more studies will be necessary to identify whether this phenomenon drives breast cancer progression in humans. If this is found to be the case, then these pathways could be considered for therapeutic intervention.

## Conclusion

In conclusion, we have developed a mammary carcinogenesis model in Nrf2 KO mice. Treatment with the carcinogen, DMBA, resulted in the formation of premalignant lesions in mouse mammary glands, but no differences in these premalignant lesions were observed between WT and Nrf2 KO mice. Instead, Nrf2 KO mice developed rapidly growing, aggressive mammary carcinomas that were larger than the WT mammary carcinomas. Although there was no difference in overall survival between WT and KO mice, there was a significant decline in mammary tumor-free survival in the KO mice. The active forms of NF-κB and β-catenin were significantly elevated ~2-fold in the KO mammary carcinomas. Real-time quantitative pcr analysis suggested that Casp3 was downregulated in the KO mammary carcinomas. Many other lesions were observed in the tumor study, particularly lung adenomas, which were more predominant in the Nrf2 KO mice. We conclude that Nrf2 KO mice were more susceptible to DMBA-induced mammary carcinogenesis, particularly in terms of tumor size, rate of growth, and mammary tumor-free survival. Key factors involved in the rapid growth of these carcinomas remain to be determined. Identification of factors responsible for the rapid tumor growth rate may provide insight into human breast cancer.

## Abbreviations

AUR: Auraptene; BRDU: 2-Bromodeoxyuridine; CDNB: 1-chloro-2,4-dinitrobenzene; DCNB: 1,2-dichloro-4-nitrobenzene DMBA: 7,12-dimethylbenz[a]anthracene; DVL: Dissheveled; FZD: frizzled; GSH: glutathione (reduced form); GST: Glutathione S-transferase; NQO1: NAD(P)H quinone oxidoreductase; HO-1: heme oxygenase 1; MPO: medroxyprogesterone acetate.

## Competing interests

The authors report no competing interests. All authors have read and agreed to the final version of the manuscript.

## Authors' contributions

LB conducted the majority of the premalignant study, wrote the first draft of the manuscript, and presented her poster at the Experimental Biology meeting in Anaheim, 2010. MP bred and genotyped the mice, conducted the comparison study (comparing the two different sources of AUR), maintained the body weight and caliper charts, and assisted in every aspect of the study, including all the necropsies. MM assisted in necropsies, histology, and generation of the stable cell lines. HB assisted in making tissue lysates, conducted all the NF-κB studies, aided in the discussion and interpretation of the q-pcr gene arrays and β-catenin analyses, and compiled Figure 1. CC processed the whole mounts and assisted in data collection and western blots. MB conducted the oxyblots. KI and MY provided the mice and intellectual input during the design and interpretation of the study. EO assisted in supplying the criteria for euthanasia, and with the assistance during necropsies and identification of lesions. EO also provided advice during the initial planning of the study design. RS and JM assisted in the initial planning of the study design, and in statistical interpretation of the results. KP provided the expertise on the β-catenin pathway and conducted those western blots. SZ evaluated all of the whole mounts and histopathology. SZ also provided the photomicrographs and valuable intellectual input during and after the study. HEK was responsible for the overall idea, design, and execution of the study, and leading the research team to the results provided in the manuscript. HEK also assisted LB in finalizing the manuscript.

## Authors' information

LB, B.S., is currently a M.S. student. MP, B.S., is the laboratory manager and student in nursing school. HB, CC, and MB were high-school students. KI, M.D., Ph.D., and MY, M.D., Ph.D. are both Professors in Japan. MM, Ph.D., is a Professor. EO, DVM, is the Associate Director of the Department of Veterinary Medicine. RS, Ph.D. and J.M., Ph.D. are both statisticians. KP is an Assistant Professor. SZ, M.D., Ph.D., is a pathologist. HEK is an Associate Professor and the Director of the Breast Cancer Focus Group at the Feist-Weiller Cancer Center.

## Pre-publication history

The pre-publication history for this paper can be accessed here:

http://www.biomedcentral.com/1471-2407/10/540/prepub

## Supplementary Material

Additional file 1 **Additional Figures and Tables**. Figure S1: Liver cytosolic GST activity in the comparison study using CDNB and DCNB as substrates. Female Nrf2 wild-type or knockout mice (age 6-8 weeks old) were administered vehicle (corn oil, 0.1 ml per 25 g bw) or auraptene (150 mg/kg bw) once a day for 3 consecutive days. At 24 h after the final dose, mice were sacrificed and livers removed. Liver cytosolic fractions were obtained by differential centrifugation. Glutathione S-transferase activity (using either CDNB or DCNB as substrates) was analyzed by the method of Habig. Figure S2. Liver cytosolic GST activity in the premalignant study using CDNB and DCNB as substrates. Cont., control diet, AUR, auraptene diet *Significantly different from vehicle control group with control (AIN76A) diet at p < 0.05 (ANOVA, Fischer's PLSD test), but not in the KO mice. DMBA groups, WT mice increased in GST activities fed either auraptene or control diet vs. KO mice. Figure S3. Body weight chart from premalignant study. The group of KO mice dosed with carcinogen had a lower bw at the beginning of the study, but they caught up by the end, where there was no significant difference. Figure S4. Body weight chart for the tumor study. No major changes in body weights were observed amongst the groups in the tumor study. Body weights (mean ± SD) are plotted as a function of days on the study. Figure S5. Box plot showing no major changes in body weights were observed amongst the groups in the tumor study. Figures represent area under the curve. Table S6. Rate of growth of mammary carcinomas (linear phase) mm^3^/day. *Rate of growth was estimated by taking the difference in tumor volume from two dates (or four dates for biphasic curves) in the linear phase of growth. Some curves were wavy, so a best-fit line was drawn to estimate tumor growth. WT mice: Many tumor remained dormant for a long time or throughout the study--tumors that grew very slowly, never got big, or were only found at necropsy, 117 T3, 176 T1, 181 T1, 146 T1, 125 T3, 127 T1, etc. Average (not including slow-growing tumors) = **28 mm^3^/day**. KO mice: tumors that grew slowly, were small, or only found at necropsy: 164 T2, 184 T3, 154 T1-3, 117 T3, 176 T1, 181 T1. Average (not including slow-growing tumors) = **96 mm^3^/day**. Table S7. Necropsy data for Nrf2 mammary carcinogenesis study. "Cause of death" defined as the most likely cause of death. This includes the following: lymphomas causing illness such as breathing difficulties, internal bleeding; Tumor burden, mainly palpable tumors (mammary carcinomas, with a few adenoacanthomas, and a rare occurrence of sebaceous gland tumors, and hemangiomas); Other--either large skin papillomas, ill due to unknown origin (thin), steatosis. Values represent the percentage of mice per category means ± SE (n = 34 WT; 30, KO). **Significantly different from WT mice p ≤ 0.02. Click here for file
