# Supplementary material for: Aggressive mammary carcinoma progression in Nrf2 knockout mice treated with 7,12-dimethylbenz[a]anthracene
Source: BMC Cancer. 2010 Oct 8;10:540. doi: 10.1186/1471-2407-10-540 (PMC2964634; doi:10.1186/1471-2407-10-540)
Supplement: Additional file 1 — Additional Figures and Tables. Figure S1: Liver cytosolic GST activity in the comparison study using CDNB and DCNB as substrates. Female Nrf2 wild-type or knockout mice (age 6-8 weeks old) were administered vehicle (corn oil, 0.1 ml per 25 g bw) or auraptene (150 mg/kg bw) once a day for 3 consecutive days. At 24 h after the final dose, mice were sacrificed and livers removed. Liver cytosolic fractions were obtained by differential centrifugation. Glutathione S-transferase activity (using either CDNB or DCNB as substrates) was analyzed by the method of Habig. Figure S2. Liver cytosolic GST activity in the premalignant study using CDNB and DCNB as substrates. Cont., control diet, AUR, auraptene diet *Significantly different from vehicle control group with control (AIN76A) diet at p < 0.05 (ANOVA, Fischer's PLSD test), but not in the KO mice. DMBA groups, WT mice increased in GST activities fed either auraptene or control diet vs. KO mice. Figure S3. Body weight chart from premalignant study. The group of KO mice dosed with carcinogen had a lower bw at the beginning of the study, but they caught up by the end, where there was no significant difference. Figure S4. Body weight chart for the tumor study. No major changes in body weights were observed amongst the groups in the tumor study. Body weights (mean ± SD) are plotted as a function of days on the study. Figure S5. Box plot showing no major changes in body weights were observed amongst the groups in the tumor study. Figures represent area under the curve. Table S6. Rate of growth of mammary carcinomas (linear phase) mm3/day. *Rate of growth was estimated by taking the difference in tumor volume from two dates (or four dates for biphasic curves) in the linear phase of growth. Some curves were wavy, so a best-fit line was drawn to estimate tumor growth. WT mice: Many tumor remained dormant for a long time or throughout the study--tumors that grew very slowly, never got big, or were only found at necropsy, 117 T3, 176 [file 1471-2407-10-540-S1.PDF]

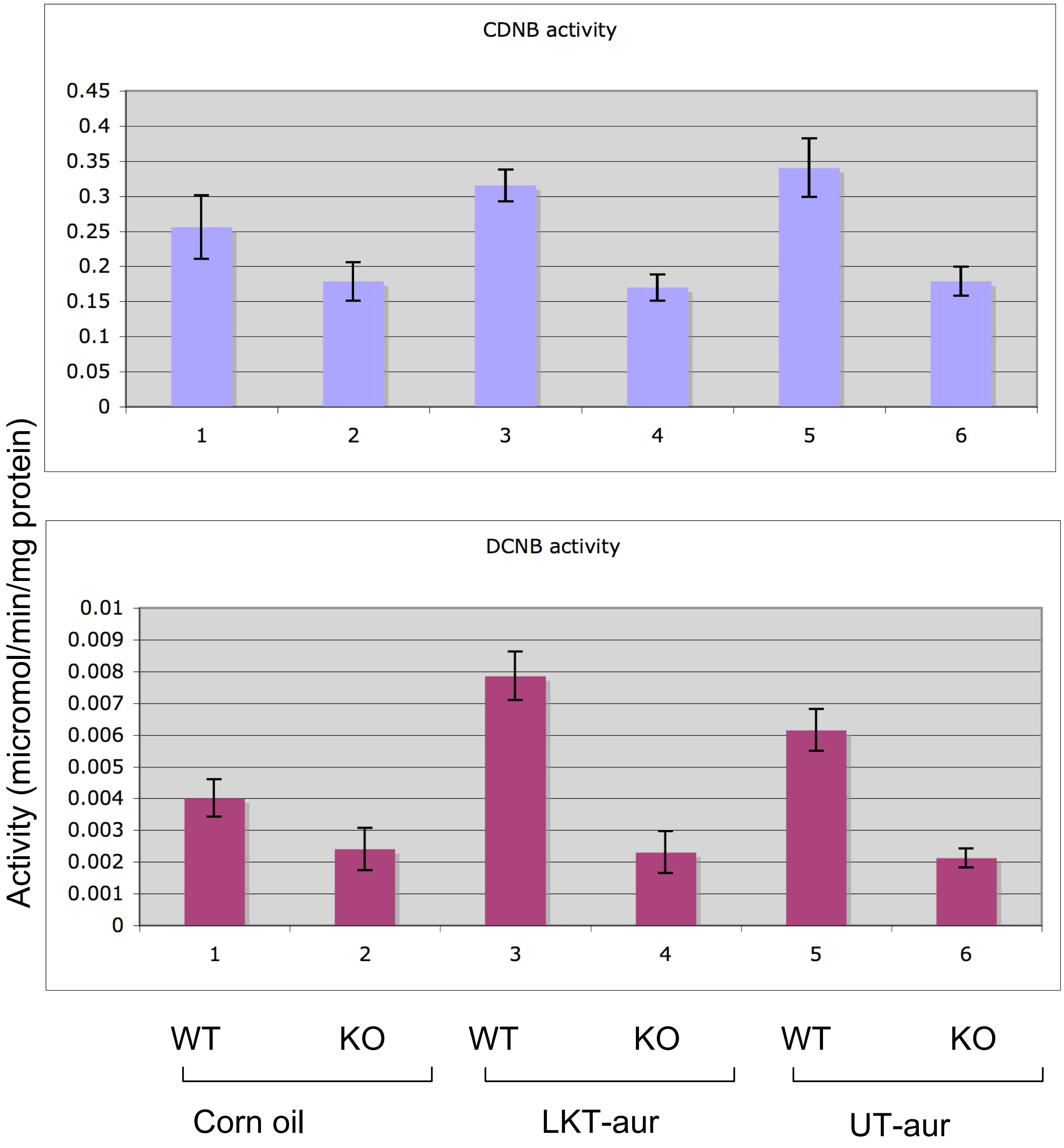

Additional File Figure S1. Liver cytosolic GST activity in the comparison study using CDNB and DCNB as substrates. Female Nrf2 wild-type or knockout mice (age 6-8 weeks old) were administered vehicle (corn oil, 0.1 ml per 25 g bw) or auraptene (150 mg/kg bw) once a day for 3 consecutive days. At 24 h after the final dose, mice were sacrificed and livers removed. Liver cytosolic fractions were obtained by differential centrifugation. Glutathione S-transferase activity (using either CDNB or DCNB as substrates) was analyzed by the method of Habig.

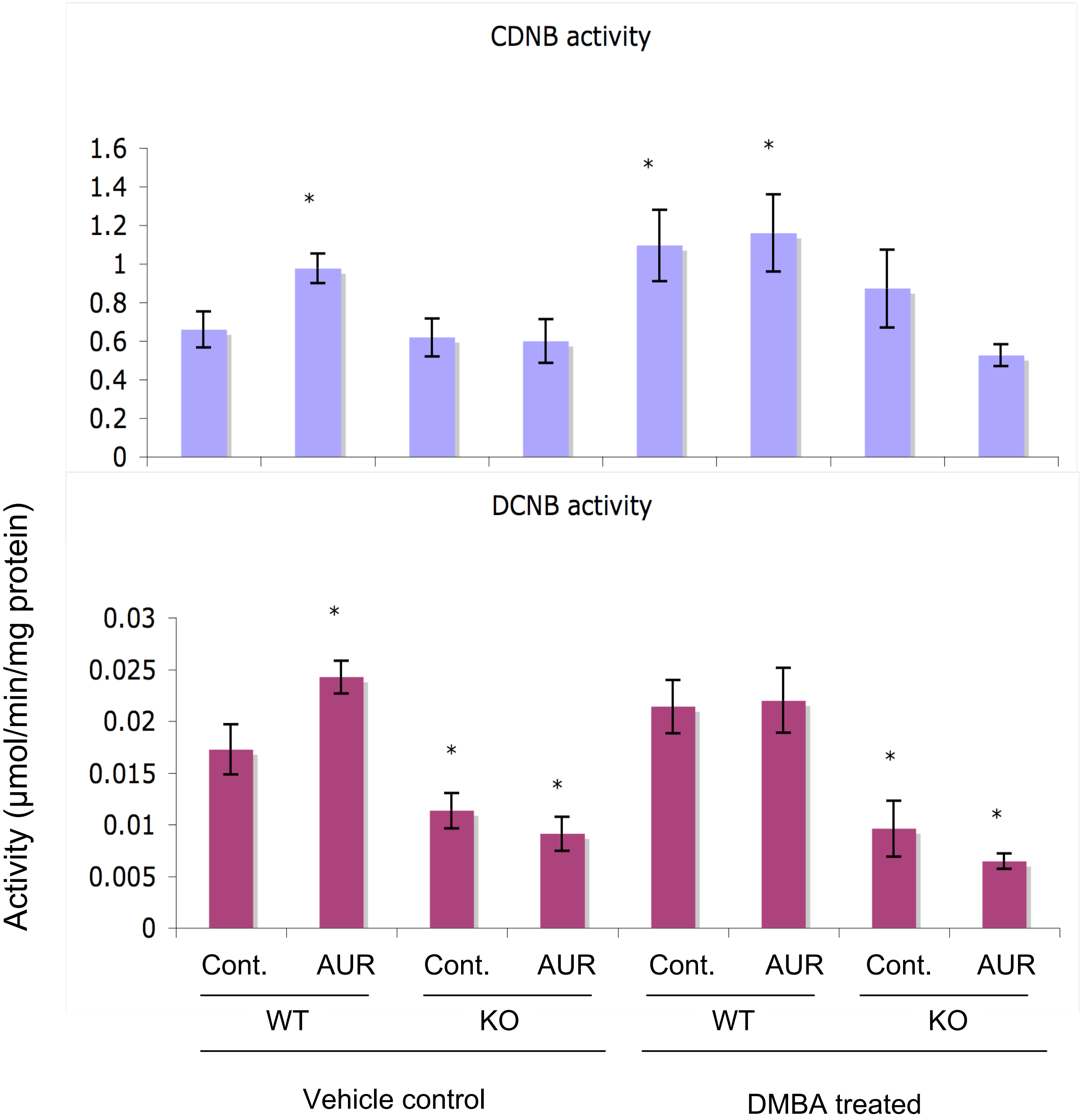

Additional File Figure S2. Liver cytosolic GST activity in the premalignant study using CDNB and DCNB as substrates. Cont., control diet, AUR, auraptene diet *Significantly different from vehicle control group with control (AIN76A) diet at $p < 0.05$ (ANOVA, Fischer's PLSD test), but not in the KO mice. DMBA groups, WT mice increased in GST activities fed either auraptene or control diet vs. KO mice.

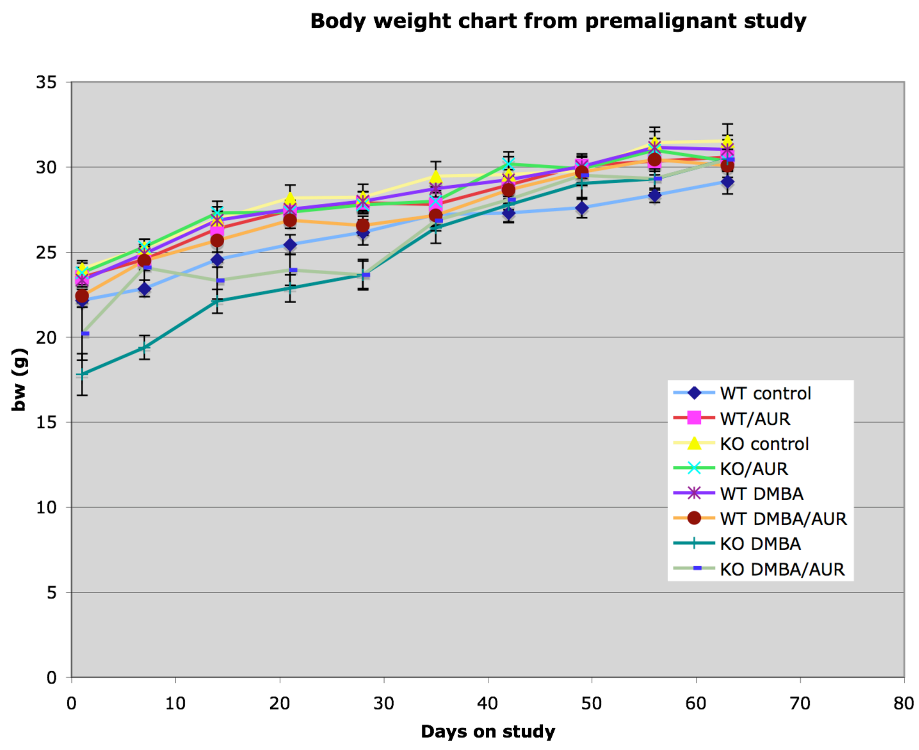

Additional File Figure S3. Body weight chart from premalignant study. The group of KO mice dosed with carcinogen had a lower bw at the beginning of the study, but they caught up by the end, where there was no significant difference.

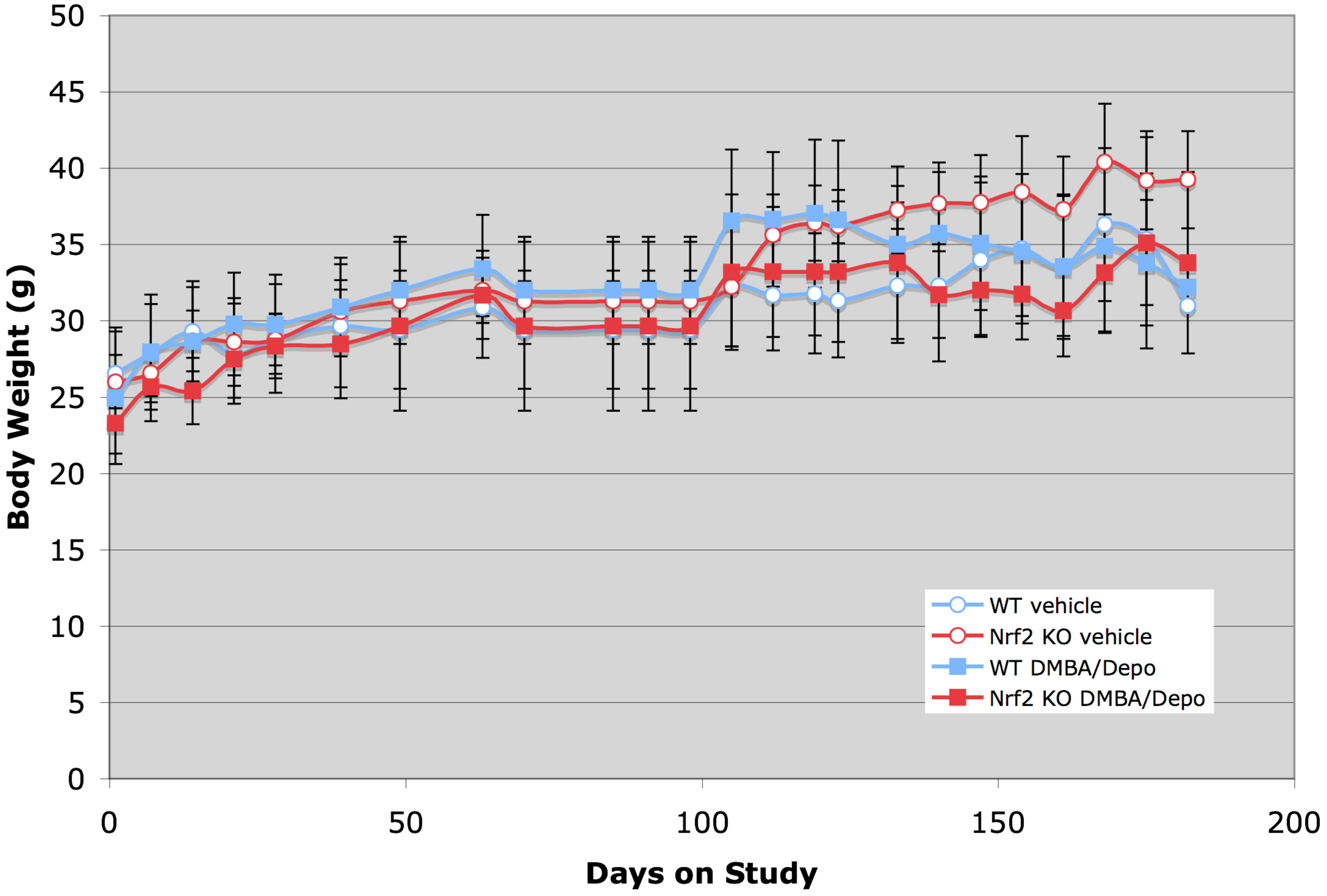

Additional File Figure S4. Body weight chart for the tumor study. No major changes in body weights were observed amongst the groups in the tumor study. Body weights (mean ± SD) are plotted as a function of days on the study.

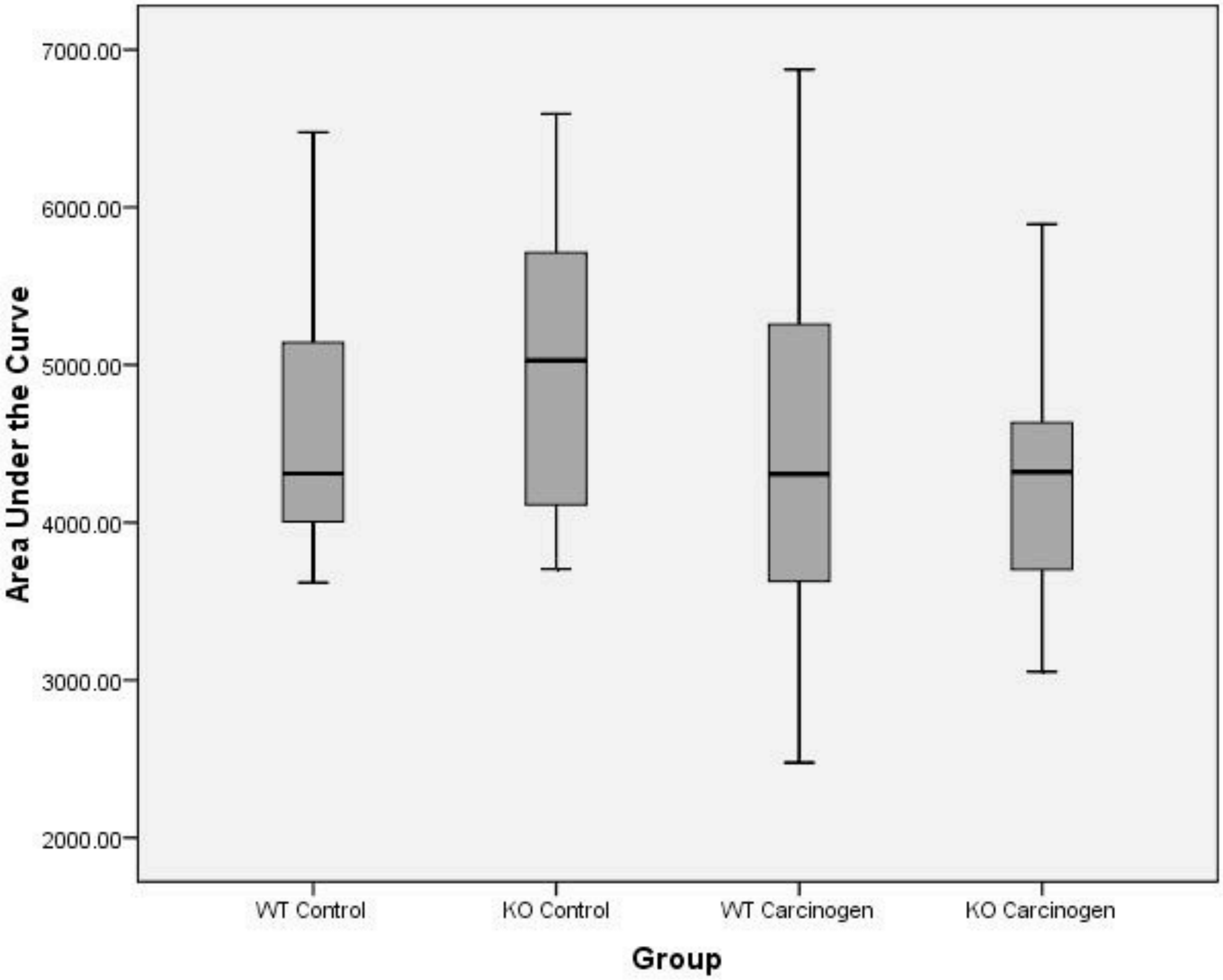

Additional File Figure S5
Box plot showing no major changes in body weights were observed amongst the groups in the tumor study. Figures represent area under the curve.

Additional File Table S6. Rate of growth of mammary carcinomas (linear phase) mm^3/day

WT mouse & tumor no.	WT rate of growth (mm^3/day)*	KO mouse & tumor no.	KO rate of growth (mm3/day)*
133 T1	8	173 T2	326
138 T1 biphasic	14, 69	173 T1	18
144 T1	45	150 T1 biphasic	74, 111
145 T1	27	153 T1	16
185 T1	12	180 T1	63-95 (avg 77)
189 T2	14	117 T2	15
125 T2	17	117 T4	18
187 T2	18	164 T1	69
188 T2 biphasic	12, 50	179 T1 biphasic	45, 311
188 T3	22	184 T1	195
191 T1 (origin of cell line)	22	151 T1 (origin of cell line)	28
187 T1	62	165 T1	34
196 T1	31		

*Rate of growth was estimated by taking the difference in tumor volume from two dates (or four dates for biphasic curves) in the linear phase of growth. Some curves were wavy, so a best-fit line was drawn to estimate tumor growth.

WT mice: Many tumor remained dormant for a long time or throughout the study—tumors that grew very slowly, never got big, or were only found at necropsy, 117 T3, 176 T1, 181 T1, 146 T1, 125 T3, 127 T1, etc.
Average (not including slow-growing tumors) = **28 mm^3/day.**

KO mice: tumors that grew slowly, were small, or only found at necropsy: 164 T2, 184 T3, 154 T1-3, 117 T3, 176 T1, 181 T1.
Average (not including slow-growing tumors) = **96 mm^3/day.**

Additional File Table 7. Necropsy data for Nrf2 mammary carcinogenesis study

Cause of death[1]	WT[5]	KO[5]
Lymphoma[2]	47 ± 9	27 ± 8
Tumor burden[3]	29 ± 8	60 ± 9**
Other[4]	24 ± 7	13 ± 6

[1] Defined as the most likely cause of death. [2] Lymphomas causing illness such as breathing difficulties, internal bleeding. [3] Tumor burden, mainly palpable tumors (mammary carcinomas, with a few adenoacanthomas, and a rare occurrence of sebaceous gland tumors, and hemangiomas) [4] Other—either large skin papillomas, ill due to unknown origin (thin), steatosis. [5] Values represent the percentage of mice per category means ± SE (n=34 WT; 30, KO). **Significantly different from WT mice $p \leq 0.02$.
